# Hepatitis C Virus Infection in Mongolia: Updated Provincial Data on Prevalence, Genotype Distribution, and Age-Specific Risk Factors

**DOI:** 10.3390/v17121602

**Published:** 2025-12-11

**Authors:** Amgalan Byambasuren, Myagmarjaltsan Baatarzorigt, Munkhtuya Otgon, Byambasuren Bat-Amgalan, Mandakhnaran Purevkhuu, Naranzul Nyamsuren, Enkh-Amar Ayush, Dashchirev Munkh-Orshikh, Khurelbaatar Nyamdavaa, Oidov Baatarkhuu

**Affiliations:** 1 Department of Health Research, Graduate School, Mongolian National University of Medical Sciences, Ulaanbaatar 14210, Mongolia; amgalan.ara@gmail.com; 2 General Hospital, Tsetserleg 6519, Mongolia; miigaanaa2126@gmail.com; 3 Soum Health Center of the Arkhangai Province, Ulaanbaatar 15141, Mongolia; hurleeb41@gmail.com; 4 Department of Administration, Mongolian National University of Medical Sciences, Ulaanbaatar 14210, Mongolia; batamgalan.b@mnums.edu.mn (B.B.-A.); mandakhnaran.p@mnums.edu.mn (M.P.); khurelbaatar@mnums.edu.mn (K.N.); 5 Department of Infectious Diseases, School of Medicine, Mongolian National University of Medical Sciences, Ulaanbaatar 14210, Mongolia; naranzul.dr@gmail.com; 6 Department of Gastroenterology, School of Medicine, Mongolian National University of Medical Sciences, Ulaanbaatar 14210, Mongolia; enkhamar.a@mnums.edu.mn; 7 National Cancer Center, Ulaanbaatar 13370, Mongolia; muugii@yahoo.com; 8 Mongolian Academy of Sciences, Ulaanbaatar 13330, Mongolia

**Keywords:** hepatitis C virus, prevalence, risk factors, Mongolia, genotype 1b, cupping therapy, unsafe injection

## Abstract

(1) Background: Mongolia has historically reported one of the highest hepatitis C virus (HCV) prevalence rates globally, with past national estimates exceeding 15%, making HCV infection a major public health priority. This study aimed to assess the prevalence, genotype distribution, and risk factors of HCV infection among residents of Arkhangai Province. (2) Methods: This population-based cross-sectional study was conducted in 2022 including 2304 individuals aged 0–80 years. Serum samples were tested for anti-HCV antibodies using ELISA and for HCV RNA using PCR. Positive samples were genotyped, and demographic and exposure data were analyzed using logistic regression to identify independent risk factors. (3) Results: The prevalence of anti-HCV antibodies was 12.0%, and HCV RNA positivity was 7.16%. Infection increased significantly with age (*p* < 0.001) and was higher among females (14.6%) than males (8.4%). Genotype 1b predominated (98.2%), followed by 1a (1.2%) and 2 (0.6%). Several exposures showed strong associations with HCV infection in univariate analysis, including cupping therapy (OR 2.37, 95% CI 1.71–3.28), shared razor use (OR 2.39, 95% CI 1.59–3.60), cosmetic procedures (OR 1.70, 95% CI 1.11–2.45), and unsafe injections (OR 2.06, 95% CI 1.40–3.02). In multivariable analysis, four exposures remained independently associated with HCV infection: cupping therapy (adjusted OR 1.89, 95% CI 1.32–2.70), shared razor use (adjusted OR 1.98, 95% CI 1.24–2.89), cosmetic procedures (adjusted OR 1.62, 95% CI 1.39–2.24), and unsafe injections (adjusted OR 1.84, 95% CI 1.19–2.83). (4) Conclusions: HCV infection remains prevalent, particularly among older adults and women. Genotype 1b continues to predominate, indicating that the viral genetic distribution has remained largely unchanged over the past decade. Continued education, safe injection practices, and regulation of traditional and cosmetic procedures are essential to reduce HCV transmission and support Mongolia’s elimination goals. These findings highlight the need for comprehensive prevention strategies addressing both unsafe traditional/medical practices and the rapidly expanding cosmetic and aesthetic service sector.

## 1. Introduction

Hepatitis C virus infection remains a major global health concern and one of the leading causes of chronic liver disease, cirrhosis, and hepatocellular carcinoma. According to the World Health Organization’s 2024 Global Hepatitis Report, an estimated 50 million people are living with chronic HCV infection worldwide, with approximately 2.2 million new infections in 2022 [1,2]. Each year, around one and a half million people become newly infected, and more than two hundred and ninety thousand deaths are attributed to complications such as liver failure and hepatocellular carcinoma. The global distribution of HCV infection is highly heterogeneous. High-prevalence regions such as West Africa, Eastern Europe, and Central Asia report rates exceeding 2.5%, whereas most Western European and North American countries report rates below 1% [1,2]. Such differences are largely influenced by variation in healthcare quality, infection control practices, and access to screening and antiviral therapy [3,4,5].

Mongolia has long been recognized as one of the countries most severely affected by viral hepatitis, including hepatitis B and hepatitis C. Liver cancer is the leading cause of cancer-related mortality in Mongolia, and the majority of these cases are associated with chronic hepatitis B or C infection [6,7]. Hepatitis C infection in Mongolia is notable not only for its high prevalence but also for the dominance of a single viral genotype, 1b [8]. A landmark nationwide study conducted between 2003 and 2005 by Oidov Baatarkhuu and colleagues demonstrated that 15.6% of the general Mongolian population was positive for anti-HCV antibodies and 11% for HCV RNA, with 98.8% of cases belonging to genotype 1b [9]. That study provided the first molecular epidemiologic baseline for the country and underscored the urgent need for comprehensive control strategies. Since that time, infection prevention has improved markedly through the introduction of single-use syringes, stricter sterilization standards, and the mandatory screening of all blood donors. However, HCV infection continues to pose a major public health burden, especially in rural areas where access to modern healthcare is limited and traditional practices remain common [1,8,9]. Despite these national improvements, recent population-based data from rural provinces remain scarce, underscoring the need for updated local epidemiologic assessments. HCV RNA-positive individuals can now be effectively treated with direct-acting antiviral (DAA) regimens, which achieve sustained virologic response rates exceeding 95%, offering the possibility of population-level elimination.

Arkhangai Province, situated in central Mongolia, is a region with a mix of urban and rural populations, many of whom continue to rely on traditional medicine and community-based healthcare practices. Earlier reports suggested that HCV prevalence in Arkhangai Province may exceed that of neighboring regions [6]. Yet, no comprehensive study has been undertaken in recent years using molecular diagnostic methods. The study aimed to contextualize current findings within Mongolia’s historical national epidemiology and to identify potential regional shifts relative to earlier baseline data.

## 2. Materials and Methods

### 2.1. Study Design and Participants

The study employed a community-based, cross-sectional design and was conducted among apparently healthy individuals (defined as individuals without self-reported chronic illness, acute symptoms, or known infectious disease at enrollment) aged between 0 and 80 years residing in both the provincial capital and eight randomly selected soums. A multistage random sampling approach was used to ensure representativeness of both urban and rural settings. In the first stage, the province’s administrative units were listed, and nine clusters were randomly selected. Households were selected using simple random sampling generated through a computerized random-number list. All permanent residents of selected households who had lived in the area for at least six months and who consented to participate were included in the study. Individuals with known chronic liver disease under active treatment were excluded to avoid bias related to prior diagnosis or treatment. In total, 2304 participants were enrolled, including 973 men and 1331 women.

The study was conducted following the ethical standards of the Declaration of Helsinki and was approved by the Medical ethical committee of the Mongolian National University of Medical Sciences (Approval No: 2022/Z-04, dated 29 April 2022). All participants provided informed consent for both the initial screening phase and the extended follow-up period, with strict adherence to data privacy and patient confidentiality.

### 2.2. Data Collection

Data were collected through face-to-face interviews conducted by trained public health nurses using a structured questionnaire. The questionnaire included items on demographic characteristics, medical history, previous blood transfusions, dental procedures, surgical operations, cupping therapy, acupuncture, cosmetic treatments, tattooing, and other exposures that could potentially lead to bloodborne infection. Participants were also asked about personal hygiene behaviors, such as razor sharing and the use of non-medical injections. Interviews were carried out in private settings to ensure confidentiality and accuracy.

Venous blood samples of five to ten milliliters were collected from each participant by certified nurses using sterile, single-use butterfly needles. Samples were placed in vacuum tubes containing clot-activating gel, allowed to coagulate, and centrifuged at the local health facility to obtain serum. The sera were stored at 2–8 °C and transported daily to the laboratory of the Arkhangai Provincial Central Hospital for serological screening and storage prior to molecular analysis.

Serological screening for anti-HCV antibodies was performed using the Architect Anti-HCV chemiluminescent microparticle immunoassay (Abbott Diagnostics, Abbott Park, IL, USA), which has a reported sensitivity of 99.1% and specificity of 99.6%. All initially reactive specimens were retested using the same assay, and results are therefore reported as “repeat reactive” rather than confirmed positive. HCV RNA testing was conducted using the Roche COBAS AmpliPrep/COBAS TaqMan HCV Test, v2.0 (Roche Molecular Diagnostics, Mannheim, Germany), a real-time PCR assay with a lower limit of detection of 15 IU/mL and a dynamic quantification range of 15–10^7^ IU/mL. In remote district facilities where centralized PCR capacity was unavailable, RNA testing was complemented using the GeneXpert^®^ HCV Viral Load Assay (Cepheid, Sunnyvale, CA, USA), which has a detection limit of 10 IU/mL. HCV genotyping was performed for RNA-positive specimens using the Roche COBAS^®^ 4800 HCV GT assay, which differentiates genotypes 1–6 based on real-time PCR amplification of conserved genomic regions.

### 2.3. Statistical Analysis

Continuous variables were summarized as means and standard deviations or as medians with interquartile ranges, depending on their distribution, and compared using Student’s *t*-test or the Mann–Whitney U-test as appropriate. Categorical variables were expressed as frequencies and percentages and analyzed using chi-square tests.

Logistic regression analysis was used to identify risk factors associated with HCV infection. In the univariable model, crude odds ratios were calculated for each potential risk factor. Adjusted odds ratios (aORs) were estimated using multivariable logistic regression. Variables with a *p*-value less than 0.10 were included in the multivariable model to adjust for potential confounding effects. Age and sex were retained in all multivariable models regardless of significance. Multicollinearity was assessed using variance inflation factors, all < 2.0, indicating no concerning intercorrelation.

All statistical analyses were performed using SPSS software, version 27.0. A significance threshold of *p* < 0.05 was applied to determine statistical significance.

## 3. Results

A total of 2304 individuals aged 0–80 years were included in the study. The majority of participants were between 21 and 50 years of age, while only a small proportion were older than 60 years. Females comprised 57.8% of the study population, slightly more than males (42.2%). More than half of the participants (57.0%) resided in rural soums, and 43.0% were from the provincial capital. This distribution provided adequate representation of both sexes and residential settings for subsequent analysis of HCV prevalence and associated risk factors (Table 1).

Molecular testing identified genotype 1b as the predominant strain, accounting for 98.2% of all RNA-positive cases. Minor proportions of genotype 1a (1.2%) and genotype 2 (0.6%) were also detected, while genotypes 3–6 were not observed in this population. These findings indicate that genotype 1b continues to be the dominant circulating strain in the region, consistent with earlier national reports.

**Figure 1:**
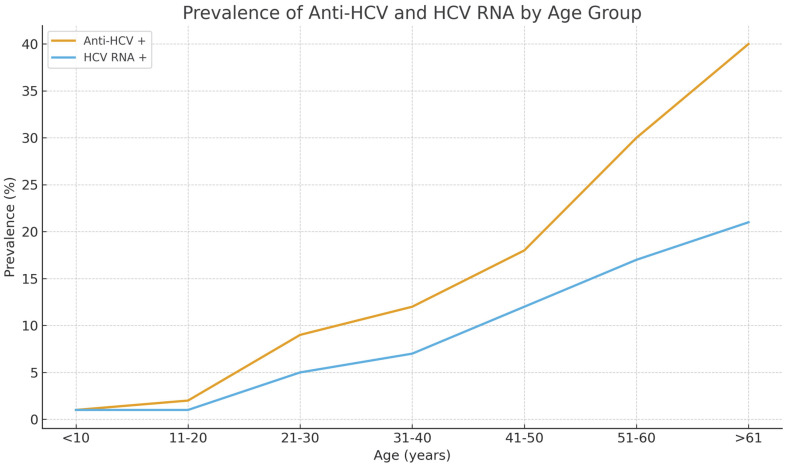
Age-specific prevalence of anti-HCV antibodies and HCV RNA among 2304 residents of Arkhangai Province, Mongolia.

Among all participants, the overall prevalence of anti-HCV antibodies was 12.0%, and HCV RNA positivity was 7.16%. Approximately 40% of anti-HCV–reactive individuals were RNA-negative, a pattern consistent with resolved infection through spontaneous clearance or prior antiviral therapy. The prevalence of infection increased significantly with age (*p* < 0.001). Anti-HCV positivity was observed in only 1.2% of individuals under 20 years of age but rose to 7.7% among those aged 31–40 years, 14.9% in the 41–50-year group, and exceeded 40% in participants aged over 61 years. A similar trend was evident for HCV RNA, suggesting that both past exposure and active infection accumulated with increasing age (Figure 1). Among anti-HCV repeat reactive individuals, 73.2% of males and 53.8% of females were RNA positive (*p* < 0.01). The lower RNA-positivity proportion in females is consistent with either higher false-reactivity or greater rates of spontaneous or treatment-induced viral clearance. A total of 277 participants were anti-HCV repeat reactive, of whom 165 (59.6%) were HCV RNA positive.

Sex-stratified analysis revealed higher prevalence among females (14.6%) compared with males (8.4%), and this difference remained statistically significant after adjusting for age. After age adjustment, female sex remained significantly associated with HCV infection (aOR 1.82; 95% CI 1.31–2.49; *p* < 0.01). The rate of HCV RNA positivity was slightly higher among women (7.9%) than men (6.2%), although this difference was not statistically significant (*p* = 0.121). These findings, together with the higher anti-HCV seroprevalence in women, suggest that females may experience greater lifetime exposure to potential risk factors such as medical or cosmetic procedures (Table 2). Overall, both anti-HCV antibody and HCV RNA positivity showed a clear age-dependent increase in both sexes. Among males, anti-HCV prevalence rose from 0.6% in the 0–10-year group to 20.8% in those aged 71 years and older, while RNA positivity increased from 0% to 16.7% across the same range. Among females, the pattern was even more pronounced, with anti-HCV positivity increasing from 0.7% to 54.5%, and RNA positivity from 0.7% to 24.2%. Chi-square analysis confirmed a statistically significant association between age and HCV positivity in both sexes (*p* < 0.001). These findings support an age-accumulative pattern of infection, with females showing higher prevalence at nearly all age categories (Table 2).

Table 2 presents age- and sex-specific prevalence patterns of HCV infection. Building on these findings, we next examined potential behavioral and medical risk factors that may explain the observed differences.

Table 3 summarizes the prevalence of potential risk factors for HCV infection by anti-HCV status and sex. Several exposures demonstrated clear sex-related differences. Cosmetic procedures, cupping therapy, injections performed outside medical facilities, and tattooing were notably more common among females, whereas shared razor use was reported almost exclusively by males (53.3% vs. 1%). In contrast, other exposures showed comparable distributions between males and females, with no statistically significant differences.

Table 4 further stratifies these exposures by age group to highlight potential age-related patterns in HCV RNA positivity. Younger participants (<35 years) demonstrated higher exposure to dental procedures and minor surgical interventions, whereas individuals ≥ 55 years showed a substantially higher prevalence of injections under non-medical conditions (60%) and cupping therapy (56%). Although some exposures displayed marked variation across age strata, these analyses were descriptive, and formal interaction tests did not identify statistically significant age-exposure interactions. Accordingly, differences observed between age groups should be interpreted with caution. Because several procedure-related exposures were of particular interest, Supplementary Table S1 provides a detailed breakdown of HCV RNA positivity by exposure status, including the total number of individuals with and without each exposure and the proportion who were HCV RNA-positive. This supplementary table complements the main analyses by offering a more granular quantitative assessment of the distribution of specific risk procedures.

In Supplementary Table S1, several exposures showed notably higher HCV RNA positivity among individuals who reported the exposure compared with those who did not. The strongest associations were observed for shared razor use (14% vs. 6.4%, *p* = 0.001), injections administered outside medical facilities (12.3% vs. 6.4%, *p* = 0.001), and cupping therapy (12.1% vs. 5.5%, *p* = 0.001). Cosmetic procedures also demonstrated a modest but statistically significant increase in RNA positivity (10.5% vs. 6.5%, *p* = 0.006). In contrast, exposures such as surgical procedures, acupuncture, tattooing, and blood transfusion did not show statistically significant differences. These pooled results complement the sex- and age-stratified analyses by confirming that certain procedure-related exposures—particularly those performed in informal settings-remain consistently associated with a higher likelihood of HCV RNA positivity.

Risk factor analysis revealed several exposures independently associated with HCV infection. To ensure clarity, the narrative presents these factors in descending order of effect size based on the multivariate model. Among the evaluated exposures, shared razor use, cupping therapy, and unsafe injections showed the strongest independent associations with HCV infection, with adjusted ORs ranging from 1.84 to 1.98 (Table 5). Shared razor use also showed a substantial association, with exposed individuals demonstrating almost twice the odds of infection compared with non-exposed participants (aOR 1.98, 95% CI 1.24–2.89, *p* < 0.001). Cosmetic procedures, including facial treatments, ear piercing, and eyebrow tattooing, were likewise significantly associated with HCV infection (aOR 1.62, 95% CI 1.39–2.24, *p* < 0.001), while injections administered outside medical facilities remained an important contributor to infection risk (aOR 1.84, 95% CI 1.19–2.83, *p* < 0.01). In contrast, other exposures such as tattooing, acupuncture, dental procedures, minor surgical procedures, surgical operations, and blood transfusion demonstrated elevated crude odds ratios in univariate analysis but did not retain statistical significance after adjustment for confounding variables. This pattern suggests that while these exposures may contribute to HCV transmission, their independent effects are weaker when co-occurring risk factors are considered (Table 5). Collectively, these findings indicate that both traditional practices (such as cupping therapy and non-medical injections) and modern cosmetic interventions play a significant role in sustaining HCV transmission within the population. These results, together with the age-related rise in infection prevalence and the persistence of genotype 1b, reinforce the conclusion that HCV remains endemic, with ongoing transmission linked to preventable medical and non-medical procedures.

These regression findings were consistent with patterns observed in Supplementary Table S1, where exposures such as non-medical injections, cupping therapy, cosmetic procedures, and shared razor use also showed the highest overall HCV RNA positivity compared with non-exposed groups.

When stratified by age, the patterns of risk exposure varied substantially across groups (Supplementary Table S2). Among participants younger than 35 years, cosmetic procedures and shared razor use emerged as the main contributors to HCV infection, both showing modest but statistically significant associations (adjusted OR 1.1–1.3, *p* < 0.05). These findings suggest that younger individuals are more likely to contract HCV through lifestyle-related or cosmetic exposures rather than medical interventions. In the 35–54-year age group, a broader range of exposures was implicated. Cosmetic procedures (aOR 1.4, *p* < 0.05), cupping therapy (aOR 1.1, *p* < 0.05), and non-medical injections (aOR 1.3, *p* < 0.05) were independently associated with HCV infection. Additionally, prior surgical operations and tattooing demonstrated elevated odds in univariate analysis, indicating possible cumulative medical or cosmetic exposure during middle age. For participants aged 55 years and older, traditional and medical practices were the dominant risk factors. Cupping therapy (aOR 1.1, *p* < 0.05), acupuncture (aOR 1.0, *p* < 0.05), and injections outside medical settings (aOR 1.9, *p* < 0.01) showed significant associations with HCV positivity, suggesting that unsafe medical or folk practices prevalent in earlier decades continue to contribute to infection risk among older adults. Taken together, these results indicate that the predominant transmission routes for HCV differ by age cohort—cosmetic and grooming-related exposures in younger populations, and traditional or healthcare-associated procedures in older groups.

## 4. Discussion

This population-based study provides an updated and detailed overview of the prevalence, genotype distribution, and associated risk factors of hepatitis C virus (HCV) infection among residents of Arkhangai Province, Mongolia. The overall anti-HCV seroprevalence of 12.0% and RNA positivity of 7.16% indicate that HCV infection remains a significant public health concern in this region, although the rate appears slightly lower than national estimates from previous decades. The predominance of genotype 1b, detected in 98.2% of RNA-positive cases, further confirms the genetic homogeneity of circulating strains in Mongolia. These findings are consistent with earlier reports and underscore that despite ongoing national screening and treatment programs, HCV transmission persists, especially among middle-aged and older adults [10,11].

The prevalence of HCV observed in this study aligns with previous national and regional data. Earlier nationwide surveys reported an HCV prevalence of approximately 15.6% [9], while more recent investigations among adults aged 10–64 years found a lower rate of 9.4% [12]. The current study’s estimate of 12.0% among a representative sample of 0–80-year-old residents from both rural and urban settings suggests that HCV infection continues to affect a considerable portion of the Mongolian population. The lower prevalence compared with the 2003–2005 national estimate likely reflects long-term improvements in infection control and increasing DAA treatment coverage. Conversely, the higher prevalence relative to a recent 9.4% estimate may reflect geographic variation and the ongoing use of traditional and informal procedures in rural settings [1,6]. The age-specific pattern revealed a clear increasing trend in seroprevalence with advancing age, rising from less than 2% among individuals under 20 years to over 40% among those aged 61 years and above. This gradient reflects cumulative lifetime exposure to parenteral risks and the historical context of medical and traditional practices in Mongolia. Similar trends have been reported in neighboring Asian and Eastern European countries, where unsafe medical procedures and cultural practices such as cupping or acupuncture were more common before modern infection control standards were widely implemented [13,14,15,16]. Because anti-HCV screening assays may yield false-reactive results, the interpretation of our findings prioritizes HCV RNA positivity as the definitive indicator of active infection. The RNA-based prevalence observed in this study aligns with national data showing that Mongolia has the second-highest HCV RNA prevalence globally, surpassed only by Egypt, although driven by distinct epidemiologic and historical exposures. Consistent with prior molecular studies, genotype 1b remained dominant in our sample, mirroring patterns reported in adjacent regions of south Siberia and northeastern China [17,18,19].

The sex-specific analysis showed that HCV infection was more prevalent among women than men, a pattern that has also been reported in several national and regional studies [20,21]. The higher infection rate among females may reflect greater healthcare utilization, including gynecological and obstetric procedures, as well as higher participation in cosmetic and aesthetic treatments [20,21]. It may also be related to sociocultural factors that increase women’s exposure to household-level transmission risks, such as sharing personal care items or undergoing informal injections and beauty treatments. The higher infection prevalence among women aligns with their markedly greater exposure to cosmetic procedures and non-medical injections, both of which emerged as strong independent predictors. These observations emphasize the need for targeted awareness and prevention strategies that consider gender-specific behavioral and healthcare patterns [20,21]. An important nuance in our findings is the discrepancy between anti-HCV seroprevalence and HCV RNA positivity according to sex. Although women showed a significantly higher rate of anti-HCV reactivity than men, active HCV infection—as defined by RNA positivity—did not differ significantly between sexes. Among repeat-reactive individuals, the proportion who were RNA-positive was substantially lower in women than in men, suggesting a higher frequency of false-reactive anti-HCV results and/or greater viral clearance among females. These observations are consistent with reports from other settings that describe sex-related differences in immune response and spontaneous HCV clearance, and they emphasize the need to base epidemiologic estimates and risk-factor analyses on HCV RNA rather than anti-HCV alone. From a public health perspective, our data support the use of confirmatory RNA testing, particularly among women, to avoid overestimating true infection burden and to better target treatment and prevention efforts.

The strong predominance of genotype 1b in the present study corroborates previous molecular findings from Mongolia and other Central Asian populations. Genotype 1b has been associated with parenteral transmission routes and a higher risk of progression to liver cirrhosis and hepatocellular carcinoma [22,23]. Its widespread distribution across different age groups in Arkhangai Province indicates long-term endemic circulation rather than recent introductions of diverse genotypes. The low occurrence of genotypes 1a and 2 suggests minimal international introduction of new strains, likely reflecting limited migration and population movement in this rural region. The persistence of genotype 1b dominance has important implications for antiviral treatment planning, as direct-acting antiviral (DAA) regimens vary slightly in efficacy by genotype [11,24,25].

Risk factor analysis revealed several significant exposures independently associated with HCV infection [26,27]. Cosmetic procedures were identified as an important source of transmission, with individuals who had undergone such procedures being more than twice as likely to be infected compared to those without such exposure. This association remained significant even after adjustment for confounding factors. The finding highlights the potential risk of transmission through inadequately sterilized tools used in cosmetic salons and beauty clinics, where infection control protocols may be insufficiently enforced. Similar associations have been reported in recent studies from Turkey, Egypt, and China, indicating that cosmetic and personal grooming practices can contribute to ongoing community transmission, particularly in settings with limited regulatory oversight [28,29,30].

Traditional therapeutic practices such as cupping therapy and acupuncture were also strongly associated with HCV infection. These procedures often involve minor skin incisions, reuse of instruments, or improper sterilization, creating opportunities for bloodborne virus transmission [31,32]. Cupping therapy, in particular, showed an adjusted odds ratio of 1.66 (95% CI 1.41–1.97), signifying a clear independent effect. In Mongolian traditional medicine, cupping is widely practiced both in professional and home settings, often without adequate infection control measures [9,12]. This underscores the importance of incorporating traditional practitioners into national infection prevention programs and raising community awareness regarding safe procedural standards.

Injections administered outside medical facilities were among the strongest independent risk factors, with exposed individuals being nearly twice as likely to test positive for HCV (adjusted OR 1.84, 95% CI 1.19–2.83) [26]. This finding reflects the persistent problem of unsafe injection practices in non-clinical settings, including the administration of vitamins, antibiotics, or painkillers by unlicensed providers. Such practices have been recognized globally as a major contributor to HCV transmission, particularly in low- and middle-income countries [16,33]. The World Health Organization estimates that nearly half of all injections worldwide are unsafe, and the current findings provide local evidence supporting that unsafe injection practices remain a key preventable source of HCV infection in rural Mongolia [34,35,36].

The use of shared razors was another significant risk factor, doubling the likelihood of HCV positivity. This reflects a mode of transmission linked to household-level behaviors and the sharing of personal hygiene items. While not unique to Mongolia, this finding reinforces the need for continued public education about everyday exposure risks that are often overlooked in national prevention programs [9,12].

In contrast, other exposures such as tattoos, dental procedures, and previous surgeries were not independently associated with infection after adjustment for confounders, despite showing elevated crude odds ratios. These findings likely reflect improved infection control in formal healthcare facilities in recent years, leading to a decline in transmission through medical procedures. The residual risk from informal or cosmetic procedures, however, remains notable and calls for stricter regulation and monitoring [37,38].

Age-stratified analysis further revealed that the predominant risk factors varied across different generations. Among participants under 35 years of age, cosmetic procedures and shared razor use were the most important predictors, suggesting that infections in younger adults may be linked to community or lifestyle exposures rather than traditional medical routes. For middle-aged individuals (35–54 years), a combination of cosmetic procedures, cupping therapy, and non-medical injections contributed significantly to infection risk, reflecting cumulative exposure during adulthood. In contrast, among those aged 55 years and older, cupping therapy, acupuncture, and non-medical injections were the principal risk factors, consistent with the practices common during earlier decades when sterilization standards were limited. This generational shift in exposure patterns has important implications for the design of age-specific prevention strategies, highlighting the need for both continued medical safety measures and community-level behavioral interventions [33,39,40].

Comparisons with previous Mongolian studies support the robustness of these findings. A nationwide survey conducted by the Ministry of Health and the National Center for Communicable Diseases in 2015 identified dental procedures (22.7%), home-based injections (20.5%), and surgical operations (11.4%) as major risk factors for HCV transmission. Our study, conducted nearly a decade later, shows that while healthcare-associated risks have decreased, informal and cosmetic procedures have emerged as new dominant routes. This transition underscores improvements in medical infection control but also highlights emerging challenges in the non-clinical sector [41,42,43]. Our findings reflect a clear epidemiologic transition: while infections linked to formal healthcare procedures have declined, unregulated cosmetic, traditional, and informal practices now constitute the dominant routes of transmission. This ‘changing of the guard’ has substantial policy implications, requiring prevention strategies that extend beyond hospital-based infection control.

Internationally, the observed associations are consistent with data from other low- and middle-income settings. Studies from Egypt, Pakistan, and China have documented similar shifts, where unsafe injections, barber-related exposures, and cosmetic treatments play a growing role in sustaining HCV transmission. The persistence of such preventable risk factors despite advances in treatment access suggests that elimination targets will not be achieved without comprehensive infection control measures beyond hospitals and clinics.

The strengths of this study include its large population-based design, inclusion of both rural and urban participants, and molecular confirmation of genotypes. The use of standardized questionnaires and laboratory assays enhances comparability with international data. Moreover, the stratified analysis by age and sex provides novel insights into generational and gender-specific patterns of exposure, contributing valuable evidence to the limited body of population-level HCV research in Mongolia.

However, several limitations should be acknowledged. First, the cross-sectional design limits causal inference, as exposures and outcomes were assessed simultaneously. Second, self-reported data on risk factors may be subject to recall or social desirability bias, particularly for sensitive behaviors. Third, while the study included a representative sample from Arkhangai Province, the findings may not be fully generalizable to all Mongolian regions, where healthcare access and traditional practices vary. Because individuals with previously diagnosed but untreated chronic liver disease were not excluded, some risk factor associations may be influenced by prior diagnoses. Finally, residual confounding from unmeasured variables, such as household income or educational level, cannot be excluded. Despite these limitations, the findings provide crucial evidence for public health planning. The identification of specific, modifiable risk factors—such as unsafe injections, cosmetic procedures, and traditional practices—highlights key targets for intervention. Strengthening infection control standards in both medical and non-medical settings, expanding community education on bloodborne transmission risks, and integrating traditional healers and cosmetic service providers into national health safety frameworks could substantially reduce new HCV infections. Because treatment history was not collected, we could not distinguish spontaneous viral clearance from DAA-induced cure among anti-HCV+/RNA− participants. Furthermore, excluding only those under active treatment may have allowed inclusion of previously diagnosed but untreated cases, potentially influencing risk associations.

Furthermore, age-targeted strategies may enhance the effectiveness of elimination efforts. For younger adults, campaigns focusing on safe cosmetic and grooming practices are warranted, while for older adults, health authorities should emphasize safe traditional therapies and screening for chronic HCV infection to prevent complications such as cirrhosis and hepatocellular carcinoma. Similar challenges have been described internationally, where micro-elimination efforts focus on socioeconomically vulnerable populations [44,45]. These parallels reinforce the importance of targeted outreach that addresses risks arising outside formal healthcare systems.

## 5. Conclusions

In conclusion, this study demonstrates that HCV infection remains a considerable burden in Arkhangai Province, Mongolia, with prevalence increasing with age and being higher among women. Genotype 1b continues to predominate, indicating that the viral genetic distribution has remained largely unchanged over the past decade. Unsafe injections, cosmetic procedures, cupping therapy, and shared razor use represent the main risk factors for ongoing transmission. These findings emphasize the urgent need for integrated preventive strategies that combine medical infection control, community education, and targeted screening to achieve national and global HCV elimination goals.

## Figures and Tables

**Table 1:** Characteristics of the study population.

Findings	Frequency	Percentage (%)
Age distribution		
0–10	301	13.1
11–20	334	14.5
21–30	401	17.4
31–40	382	16.6
41–50	396	17.2
51–60	301	13.1
61–70	132	5.7
≥71	57	2.5
Gender		
Male	973	42.2
Female	1331	57.8
Civilization		
City (urban)	991	43.0
Soum (rural)	1313	57.0

Data are presented as percentages (numbers).

**Table 2:** Age- and sex-specific prevalence of HCV infection among the study population.

Findings	Anti-HCV Positive, n (%)			HCV RNA Positive, n (%)		
	Male	Female	*p* Value	Male	Female	*p* Value
0–10	1 (0.6)	1 (0.7)	1.000	0 (0.0)	1 (0.7)	0.482
11–20	2 (1.1)	5 (3.1)	0.264	1 (0.6)	3 (1.8)	0.350
21–30	10 (7.0)	25 (10.2)	0.209	8 (5.1)	9 (3.7)	0.612
31–40	19 (14.2)	26 (11.4)	0.628	13 (8.7)	14 (6.0)	0.312
41–50	22 (14.9)	49 (19.7)	0.279	15 (8.8)	30 (12.0)	0.625
51–60	16 (12.5)	39 (22.5)	0.034	14 (10.1)	22 (12.7)	0.721
61–70	7 (10.5)	35 (37.2)	0.040	5 (13.2)	18 (19.1)	0.461
≥71	5 (20.8)	18 (54.5)	0.014	4 (16.7)	8 (24.2)	0.533
**Total**	**82 (8.4)**	**195 (14.6)**	**<0.001**	**60 (6.2)**	**105 (7.9)**	**0.121**

Data are presented as percentages (numbers).

**Table 3:** Prevalence of potential risk factors for HCV infection by sex among HCV RNA-positive individuals.

Findings	Male Exposed RNA+ (n)	Female Exposed RNA+ (n)	*p* Value
Cosmetic procedure	10 (6/60)	31.4 (33/105)	**0.001**
Dental procedure	80 (48/60)	69.5 (73/105)	0.099
Surgical operation	13.3 (8/60)	17.1 (18/105)	0.340
Cupping therapy	58.3 (35/60)	33.3 (35/105)	0.002
Acupuncture	23.3 (14/60)	22.9 (24/105)	0.540
Injection under non-medical conditions	10 (6/60)	30.5 (32/105)	**0.002**
Minor surgical procedure	15 (9/60)	16.2 (17/105)	0.500
Shared razor use	53.3 (32/60)	1 (1/105)	**0.001**
Tattooing	21.7 (13/60)	10.5 (11/105)	**0.043**
Blood transfusion	0 (0/60)	3.8 (4/105)	0.160

Data are presented as percentages (numbers).

**Table 4:** Prevalence of potential risk factors for HCV infection by age group among HCV RNA-positive individuals.

Findings	<35 Years Exposed RNA+ (n)	35–55 Years Exposed RNA+ (n)	>55 Years Exposed RNA+ (n)	*p* Value
Cosmetic procedure	26.7 (8/30)	35.3 (30/85)	2 (1/50)	**0.001**
Dental procedure	86.7 (26/30)	72.9 (62/85)	66 (33/50)	0.120
Surgical operation	6.7 (2/30)	18.8 (16/85)	16 (8/50)	0.290
Cupping therapy	26.7 (8/30)	40 (34/85)	56 (28/50)	**0.030**
Acupuncture	16.7 (5/30)	22.4 (19/85)	28 (14/50)	0.340
Injection under non-medical conditions	0 (0/30)	9.4 (8/85)	60 (30/50)	**0.001**
Minor surgical procedure	23.3 (7/30)	16.5 (14/85)	10 (5/50)	0.100
Shared razor use	26.7 (8/30)	16.5 (14/85)	22 (11/50)	**0.020**
Tattooing	20 (6/30)	8.2 (7/85)	22 (11/50)	0.050
Blood transfusion	0 (0/30)	0 (0/85)	8 (4/50)	**0.010**

Data are presented as percentages (numbers).

**Table 5:** Association of variables with increased HCV risk.

Variables	Association of Variables with the Presence of RNA+							
	Univariate OR	95% CI		*p* Value	Multivariate OR	95% CI		*p* Value
		Lower Bound	Upper Bound			Lower Bound	Upper Bound	
Cosmetic procedure	1.70	1.11	2.45	**<0.001**	1.62	1.39	2.24	**<0.001**
Dental procedure	1.36	0.96	1.98	0.05	1.08	0.83	1.54	0.30
Surgical operation	1.23	0.79	1.91	0.20	1.03	0.63	1.68	0.80
Cupping therapy	2.37	1.71	3.28	**<0.001**	1.89	1.32	2.70	**<0.001**
Acupuncture	1.21	0.83	1.77	0.17	0.87	0.58	1.30	0.50
Injection under non-medical conditions	2.06	1.40	3.02	**<0.0001**	1.84	1.19	2.83	**<0.01**
Minor surgical procedure (suturing)	1.43	0.93	2.25	0.07	1.20	0.76	2.00	0.30
Shared razor use	2.39	1.59	3.60	**<0.001**	1.98	1.24	2.89	**<0.001**
Tattooing	1.41	0.89	2.21	0.08	1.02	0.62	1.61	0.90
Blood transfusion	1.80	0.62	5.20	0.20	1.41	0.46	4.28	0.50

Data presented as odds ratio with 95% confidence intervals (95% CI).

## Data Availability

The data used to support the findings of this study are available from the corresponding author upon request.

## Supplementary Materials

The following supporting information can be downloaded at: https://www.mdpi.com/article/10.3390/v17121602/s1, Table S1: Distribution of HCV RNA positivity by exposure status across potential procedure-related risk factors; Table S2. Age-stratified analysis of risk factors associated with hepatitis C virus infection.
