# Supplementary material for: Hepatitis C Virus Infection in Mongolia: Updated Provincial Data on Prevalence, Genotype Distribution, and Age-Specific Risk Factors
Source: Viruses. 2025 Dec 11;17(12):1602. doi: 10.3390/v17121602 (PMC12737555; doi:10.3390/v17121602)
Supplement: Supplementary file 1 [file viruses-17-01602-s001.zip › viruses-3994465-supplementary.pdf]

Supplementary Table S1. Distribution of HCV RNA positivity by exposure status across potential procedure-related risk factors

Exposure	Exposed N	RNA+ (n, %)	Not exposed N	RNA+ (n, %)	p-value
Dental procedures	371	39 (10.5)	1932	126 (6.5)	0.006
Surgical procedures	1550	121 (7.8)	754	44 (5.8)	0.050
Injections outside medical facilities	307	26 (8.5)	1997	139 (7.0)	0.200
Cosmetic procedures	576	70 (12.1)	1728	95 (5.5)	0.001
Cupping therapy	460	38 (8.3)	1844	127 (6.9)	0.170
Acupuncture	309	38 (12.3)	1995	127 (6.4)	0.001
Tattooing	235	33 (14.0)	2069	132 (6.4)	0.001
Shared razor use	254	24 (9.4)	2050	141 (6.9)	0.080
Blood transfusion	33	4 (12.1)	2271	161 (7.0)	0.200

Data are presented as percentages (numbers).

Supplementary Table S2. Age-stratified analysis of risk factors associated with hepatitis C virus infection.

Risk factor	Age <35 years		Age 35–54 years		Age ≥55 years	
	Univariate OR	Multivariate OR	Univariate OR	Multivariate OR	Univariate OR	Multivariate OR
Cosmetic procedure	1.3 (1.1-1.7)	1.1 (0.9-1.3)	1.7 (1.2-2.6)	1.4 (1.0-1.8)	1.5 (0.5-5.3)	0.9 (0.5-1.4)
Dental procedure	1.3 (1.08-1.5)	0.8 (0.6-1.1)	1.1 (0.8-1.3)	0.8 (0.5-1.2)	0.9 (0.7-1)	0.6 (0.4-1.1)
Surgical operation	1.7 (0.4-6.9)	0.9 (0.7-1.2)	1.4 (0.9-2.1)	0.9 (0.5-1.4)	1.1 (0.6-1.6)	0.9 (0.4-1.7)
Cupping therapy	0.5 (0.3-1.2)	0.8 (0.6-1.1)	1.5 (1.1-2.1)	1.1 (1.0-1.2)	1.4 (1-1.8)	1.1 (1.0-1.2)
Acupuncture	0.6 (0.3-1.2)	0.8 (0.3-1.4)	1.2 (1.09-1.3)	0.8 (0.5-1.4)	1.3 (0.9-1.7)	1.0 (0.8-1.2)
Injection under non-medical conditions	-	-	1.7 (1.0-2.9)	1.8 (1.0-3.3)	1.7 (1.1-2.5)	1.9 (1.3-3.2)
Minor surgical procedure (suturing)	0.4 (0.2-0.7)	0.3 (0.1-0.7)	1.1 (0.7-2.0)	0.9 (0.5-1.7)	1.2 (0.7-2.9)	0.9 (0.6-1.3)
Shared razor use	1.2 (0.9-1.3)	1.3 (1.0-1.6)	1.1 (0.8-1.4)	0.9 (0.6-1.4)	1.0 (0.9-1.1)	0.5 (0.2-1.2)
Tattooing	0.5 (0.2-0.8)	0.7 (0.3-1.2)	1.3 (0.7-1.8)	0.9 (0.4-1.7)	1.0 (0.9-1.16)	0.7 (0.3-1.5)
Blood transfusion	0.8 (0.7-0.9)	0.9 (0.8-1.0)	0.9 (0.9-1.0)	0.9 (0.8-1.0)	1.2 (0.53-2.1)	0.9 (0.2-3.2)

Data presented as odds ratio with 95% confidence intervals (95% CI).
